# Native Spirit: Development of a culturally grounded after‐school program to promote well‐being among American Indian adolescents

**DOI:** 10.1002/ajcp.12590

**Published:** 2022-02-22

**Authors:** Amanda M. Hunter, Mikah Carlos, Velia L. Nuño, Mary Jo Tippeconnic‐Fox, Scott Carvajal, Nicole P. Yuan

**Affiliations:** ^1^ Center for Health Equity Research Northern Arizona University Flagstaff Arizona USA; ^2^ Salt River Pima Maricopa Indian Community Youth Services Department Scottsdale Arizona USA; ^3^ Department of Health Promotion Sciences, Mel and Enid Zuckerman College of Public Health University of Arizona Tucson Arizona USA; ^4^ Department of American Indian Studies, College of Social & Behavioral Sciences University of Arizona Tucson Arizona USA

**Keywords:** adolescents, after‐school program, community‐based, culturally grounded, health promotion, Indigenous

## Abstract

Culturally grounded after‐school programs (ASPs), based on local cultural values and practices, are often developed and implemented by and for the local community. Culturally grounded programs promote health and well‐being for American Indian and Alaska Native (AI/AN) adolescents by allowing them to reconnect to cultural teachings that have faced attempted historical and contemporary erasure. This article is a first‐person account that describes the development and implementation of a culturally grounded ASP, Native Spirit (NS), for AI adolescents (grades 7–12) living on a Southwest urban‐based reservation. NS, a 13‐session culturally grounded ASP, was developed by an academic–community partnership that focuses on increasing cultural engagement as a form of positive youth development. Each session was guided by one to two local cultural practitioners and community leaders. The development of the NS program contributed to an Indigenous prevention science that emphasizes the positive impacts of Indigenous culture and community on health and well‐being. The use of the ASP format, in partnership with the Boys & Girls Club, increased the feasibility of dissemination and refinement of the NS program by tribal communities and organizations.

## Highlights

- This study expands the literature on culturally grounded health promotion programs for youth.
- The Native Spirit program increases cultural engagement as a form of positive youth development.
- An academic–community partnership developed a culturally grounded program for Indigenous youth.
- The Native Spirit program may be refined by other tribal communities throughout the United States.

## INTRODUCTION

### Culturally grounded health promotion

American Indian and Alaska Native (AI/AN) adolescents are vulnerable to experiencing health disparities that continue into adulthood (Jacobs‐Wingo et al., 2016; A. Kelley et al., 2019; Sancar et al., 2017). Special attention has focused on AI/AN youth because of documented high rates of substance abuse (Goodkind et al., 2012; Kulis et al., 2017; Walls et al., 2020). The 2013 National Survey on Drug Use and National Health indicated that AI/AN youth used cigarettes, marijuana, and prescription drugs at a higher rate than the national average (Hilton et al., 2018; Substance Abuse and Mental Health Services Administration, 2014). Recent data from the Healing Pathways study shows that AI/AN youth substance use problems still persist, leading to disproportionately high rates of early‐onset and lifetime substance use disorder (Walls et al., 2020). Death by suicide is also a concerning issue for AI/AN youth (Suicide Prevention Resource Center, 2017). AI/ANs have elevated and increasing rates of suicide, with suicide rates peaking during adolescence. Between the ages of 15 and 25 years, AI/AN adolescents are twice as likely to die by suicide as any other racial or ethnic group (Suicide Prevention Resource Center, 2017).

Understanding and applying promotive factors, including cultural values and practices that promote health and well‐being in AI/AN adolescents, provide a viable form of intervention that has yet to be explored thoroughly. Research indicates that culturally engaged AI/AN youth are less likely to participate in risky behaviors and often exhibit enhanced self‐esteem and healthy identity formation (Division of Diversity and Health Equity, 2017; Kulis et al., 2017). The term cultural engagement is used broadly in the literature to refer to participation in local cultural ceremonies and practices, including speaking the language, cooking traditional foods, and attending community events. Taking an ecological approach, macrostructural forces, including cultural values and practices, are intertwined with mental well‐being for AI/AN communities (Bronfenbrenner, 1979; Fish & Syed, 2018). Cultural practices and teachings are passed down through generations and are present at the macrosystem level to impact all aspects of life.

**Figure 1:**
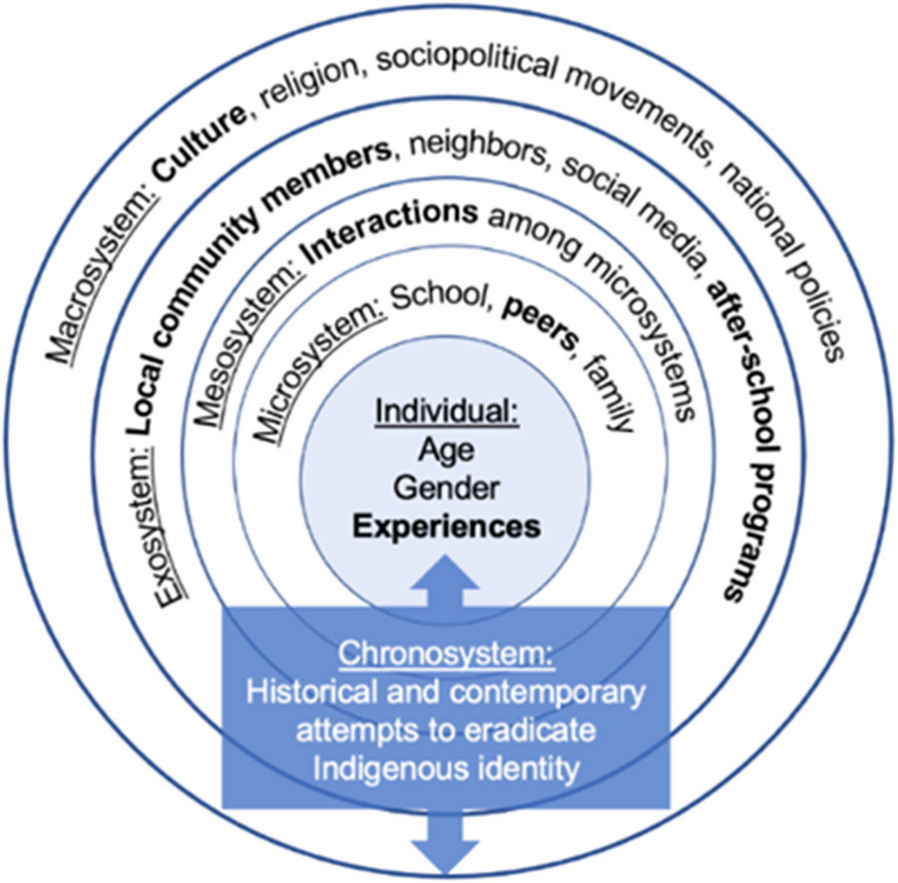
Culturally grounded after‐school programs as seen through the lens of Bronfenbrenner's Ecological Systems Model [Color figure can be viewed at wileyonlinelibrary.com]

Additionally, macro issues related to colonialism, perpetuated through time (chronosystem), have led to a blatant disregard for AI/AN health promotion approaches, including engaging in cultural practices. In the late 1800s, the US government prohibited the practice of traditional AI/AN religions and thousands of AI/AN children were forced to attend boarding schools that used corporal punishment in an effort to erase AI/AN culture (Running Bear et al., 2017, 2018). Disregard for AI/AN history, culture, and ways of life continue to this day. In August 2021, South Dakota's executive administration changed the K‐12 curriculum by erasing half of the state's AI history and culture from its textbooks (Grove, 2021). The current movement to increase cultural engagement for AI/AN adolescents as a determinant of health can increase health equity and allow AI/AN people to access their full health potential. Culturally grounded health promotion insists that cultural values (macrosystem) can be taught to individuals and groups to form healthy communities. Figure 1 demonstrates the connection between cultural engagement, history, and individual well‐being through the lens of Bronfenbrenner's Ecological Systems Model.

Several previous studies document health promotion and prevention programs for AI/AN adolescents, with various levels of cultural engagement and adaptation. For example, a continuum of approaches to developing culturally engaged interventions detail the differences between interventions with surface structure cultural adaptations, deep structure cultural adaptations, and culturally grounded interventions (Okamoto, Kulis, et al., 2014; Resnicow et al., 2000). Surface structure cultural adaptations comprise changes to intervention characteristics, including the use of local language, images that reflect the study population, and images that reflect the local scenery (Resnicow et al., 2000). Deep structure cultural adaptations incorporate specific knowledge and values of the study population within a pre‐existing intervention. For example, the Parenting in 2 Worlds curriculum is based on an intervention that was not originally designed for AI/AN communities (Kulis et al., 2016). Modifications to this parenting skills training focused on “guiding” child behavior rather than “managing” child behavior to be more in line with AI/AN child‐rearing beliefs (Kulis et al., 2016, 2020). Culturally grounded interventions increase community engagement and investment while also addressing core cultural values compared to the other interventions on the continuum (Cwik et al., 2019; Okamoto, Helm, et al., 2014; Okamoto, Kulis, et al., 2014). Additionally, culturally grounded programs are often developed by and for the local community and are rooted in specific social contexts (Cwik et al., 2019; Henderson et al., 2017).

### Culturally grounded after‐school programs

Current research uses culturally engaged after‐school programs (ASPs) to promote developmental assets in AI/AN adolescents. A recent literature review yielded 14 studies, published from 2009 to 2020, that evaluated culturally engaged ASPs for Indigenous youth (Hunter et al., 2022). Seven of these studies were culturally grounded, using local community values and practices to guide their interventions (Allen et al., 2018; D'Amico et al., 2020; Donovan et al., 2015; Hishinuma et al., 2009; A. Kelley et al., 2018; M. Kelley & Lowe, 2018; Usuba et al., 2019). Indigenous youth who participated in these culturally grounded ASPs displayed improved self‐esteem and lower levels of substance use and initiation (Donovan et al., 2015; Hishinuma et al., 2009).

Culturally grounded ASPs provide a safe space for AI/AN youth to learn and grow in a positive way and contribute to positive youth development (PYD; Durlak et al., 2007; Simpkins & Riggs, 2014). Not adequately considering the community culture in an ASP may have deleterious impacts on youth, as they may feel marginalized or targeted for engaging in their cultural interests and may therefore have lower levels of participation and/or drop out of programs entirely (Lin et al., 2016). Unfortunately, there is little peer‐reviewed research on the design, implementation, and evaluation of culturally grounded ASPs for AI/AN adolescents (Cwik et al., 2019; Okamoto et al., 2016). Documenting the development of culturally grounded ASPs is important for ensuring that the programs are responding to local community values. The emphasis in the scientific literature favoring intervention outcome studies using rigorous designs essentially marginalizes the knowledge constructed outside of academia, which has the potential to change community systems (Okamoto, 2011).

Promoting health through community change requires time and there is as much to learn from the process as there is from the final intervention outcomes (Foster‐Fishman et al., 2007). Documenting the development of a culturally grounded ASP in AI/AN communities is particularly important because of the immense variation in historical backgrounds and current cultural settings (Cwik et al., 2019; Okamoto, Helm, et al., 2014; Okamoto, Kulis, et al., 2014; Simpkins et al., 2016). For example, the development of an ASP in a Southwest rural AI community would look different than one in a Northeast urban AI community because each community speaks a different language, has a unique history, and has a different modern social context. To avoid shallow representations of AI/AN culture and improve the transparency of the research process with AI/A communities, we must describe the process of developing culturally grounded interventions. Becoming familiar with culturally grounded research processes has the reciprocal benefit of honoring tribal sovereignty in research while helping researchers avoid common pitfalls in the tribal research process. This article seeks to combat institutionalized practices of marginalizing community knowledge to the detriment of public health by sharing the development of a successful culturally grounded program.

### First‐person account case study

This article provides a detailed description of the development and implementation of a culturally grounded ASP, Native Spirit (NS), for AI adolescents living on a Southwest urban‐based reservation, the Salt River Pima‐Maricopa Indian Community (SRPMIC). The SRPMIC has agreed to be named in this article. It uses a first‐person account to detail the community and partnership contexts, program history, and the curriculum development process, and also describes program implementation and the proposed evaluation plan. Finally, this article describes challenges and recommendations to foster success for tribal communities, organizations, and researchers seeking to develop culturally‐grounded health promotion programs. Ultimately, this first‐person account contributes to the literature by detailing the amount of time and effort required to develop, implement, and evaluate a culturally grounded prevention program for AI/AN adolescents. The detailed description combats the narrative that community‐specific interventions are not rigorous and supports placing a higher value on external validity, especially for communities that have been historically underserved and are not regularly represented in research (Walters et al., 2019). This account is told from the perspective of the Principal Investigator and primary author, Amanda M. Hunter). Amanda is a member of the Pascua Yaqui Tribe and her research focuses on partnering with Native nations to develop, implement, and evaluate culturally grounded health promotion and disease prevention programs for AI/AN adolescents.

## DEVELOPING NS

The NS program and academic‐community partnership was initiated by the Boys & Girls Clubs (BGC) of Greater Scottsdale in 2015. The BGC of America is a national organization that hosts ASPs to provide a safe and educational after‐school experience for children between the ages of six and 18 years. Of the 60 BGC locations in Arizona, 13 serve AI communities; each is directed either by the larger, local BGC chapter or has started its own free‐standing location under the national organization. In 2018, BGCs in Arizona served 112,883 youth; 10% were AI (Boys & Girls Clubs Arizona Alliance, 2018). Additionally, 33% of all Arizona members were between 12 and 18 years old (Boys & Girls Clubs Arizona Alliance, 2018). Each BGC location has a branch director and at least two to four additional full‐time staff. The BGC provides space on the reservation, after‐school programming structure, periphery materials (pencils, markers, paper, and glue), and maintains regular youth attendance to assist with study recruitment, contributing knowledge to, and additional oversight for, youth participants and their interests.

In 2015, the BGC of Greater Scottsdale requested assistance from a graduate student at the University of Arizona to develop a culturally grounded program that could be used in their rural reservation location. I (A. M. H.), a master's student at the time, helped develop the program in 2015. The program was named, “Native Spirit” by the youth and BGC staff. In 2018, the BGC of Greater Scottsdale expressed interest in refining the program to fit two of their urban‐based reservation locations (SRPMIC). Although the program curriculum was refined, the name of the program was not changed. The SRPMIC prioritized adolescent and community health by providing robust opportunities for cultural engagement. As a doctoral student, I initiated the research process with SRPMIC to begin refining the program. With a small amount of funding through scholarships, I provided meals during the in‐person Community Advisory Board (CAB) meetings and invited CAB members to the recognition ceremony at the end of the program. I also organized and facilitated CAB meetings, recorded the development of the NS curriculum, and developed the NS curriculum into a comprehensive handbook detailing the program's history, curriculum, and logistics. I also coordinated NS session logistics including contacting session leaders, securing locations, and purchasing supplies and food.

### Indigenous community description

The SRPMIC is comprised of two ancestral Indigenous groups, the Akimel O'odham (River People) and the Xalychidom Piipaash (People Who Live Toward the Water). The 52,600‐acre SRPMIC, located in Maricopa County in Arizona, was established by Executive Order on June 14, 1879 (Community Research Evaluation and Development, 2018). This urban‐based reservation is now surrounded by four large municipalities in the Phoenix metropolitan area. Of the approximately 10,800 enrolled members, about half reside within the boundaries of the reservation and many others live in close proximity (Community Research Evaluation and Development, 2018). Approximately 5200 of the enrolled members are aged 24 years or younger (Community Research Evaluation and Development, 2018).

The SRPMIC Tribal Council first approved the development, implementation, and evaluation of the NS program followed by the University Human Subjects Protection Program and Institutional Review Board. The work included developing a research proposal and providing information on recruitment strategies, inclusion and exclusion criteria, and participant and data protection. I presented this information at the biweekly Tribal Council meeting to seek approval. During the meeting, members of the Council asked about plans for sustainability and dissemination of the research. The development, implementation, and evaluation of the NS program were monitored regularly by a subset of two Tribal Council members and the study's CAB.

The current study is a refinement of the original NS program for use in an urban‐based reservation community in Arizona, which implemented and evaluated the refined program. The two urban‐based BGC sites are approximately five miles apart and are both located on the reservation of the SRPMIC. Although the BGC sites are located on the reservation, we describe the area as an urban‐based reservation due to its proximity to the Phoenix metropolitan area. Both locations primarily serve the youth of the SRPMIC but also have participants who are members of other tribal communities, have mixed heritage, or do not identify as AI.

### Community Advisory Board

With the help of BGC staff, I assembled a seven‐member CAB to guide the refinement, implementation, and evaluation of NS to ensure it remained culturally grounded for the SRPMIC. I recruited CAB members from each of the partnering departments and included representatives from the BGC, Cultural Resources Department, Tribal Council, and Youth Services Department. CAB formation was informed by the CAB's purpose to guide the identification of appropriate members and decisions regarding recruitment and assessment (Yuan et al., 2019). Five CAB members were from the SRPMIC and felt comfortable guiding the process of curriculum development. These members understood local cultural values and practices and also had social capital in the community, which was necessary to recruit session leaders. Two CAB members were representatives from each BGC clubhouse, and although they were not members of the SRPMIC, they each worked in the SRPMIC for at least 10 years and had detailed knowledge of the BGC's role in the community with the local adolescents.

In‐person CAB meetings were held at the SRPMIC's tribal government complex once a month during the 6‐month refinement phase and every other month during the 4‐month implementation phase of the NS study. As a culturally grounded program, the CAB initiated the development, guided the curriculum refinement, and led all of the sessions that focused on cultural values and activities. At least 30 community members, including the CAB, were invited to review and provide feedback on the curriculum, with 10 members providing an in‐depth review before implementation. Community members who reviewed the curriculum included program session leaders and others who were invited to lead program sessions but were unable to due to timing (work or family commitments). Some community members wanted to contribute to the curriculum even if they were not able to serve as session leaders. The SRPMIC has a copy of the curriculum and can make changes as necessary as the community changes.

### NS intervention

The NS program emphasizes the importance of cultural engagement in health promotion and PYD for AI/AN youth. A PYD framework focuses on individual and community strengths, rather than individual psychopathologies, and adheres to six organizing principles (Deutsch et al., 2017). Table 1 establishes a connection between the organizing principles of PYD and the NS program.

**Table 1:** Connection between PYD and NS

PYD principle	NS principle
All youth are capable of positive development	NS is open and available to all youth in the community regardless of behavioral status in school or in the community
Settings and relationships are key to fostering s developmental trajectories	NS recruits local community members to serve as session leaders, establishing a safe environment to interact with a positive social influence
Being involved in multiple promotive settings and relationships enhances positive development	NS is housed within the youth‐serving ASPs that exist in the community, providing more promotive interactions in the after‐school setting
Strategies and tactics for promoting developmental assets can vary considerably as a function of social location	NS emphasizes the increase in cultural engagement as a necessary tactic for promoting developmental assets in the local American Indian community
Community is a key setting for fostering PYD	NS is community‐developed and community‐based
Youth are agents of their own development and significant resources for creating the kinds of relationships, contexts, ecologies, and communities that enable PYD	NS places emphasis on community values that can seek to guide youth along their paths to becoming an individual member of the community

Abbreviations: ASPs, after‐school programs; PYD, positive youth development; NS, Native Spirit.

Cultural values identified in the original program included community‐specific knowledge of language, creation stories, history, and traditions. Cultural values also included the more general values of respect, patience, growth, pride in cultural identity and community, and recognition of participation in the program. The NS program remains culturally grounded because it is based on local cultural values and practices that are indicated by the community. Although some cultural values are shared by multiple communities, the practices that are associated with each value may be different for different communities. For example, the value of respect is associated with the cultural practice of harvesting food, but the act of harvesting is different for each community depending on the geographical location, the harvesting cycle of the specific plant, and the lessons that are taught. Another example is passing creation stories from one generation to the next. Although all AI/AN communities have creation stories and stories of traditional characters (plants, animals, celestial bodies, etc.), each community has a different story that is often intricately related to the local flora, fauna, and geographical landscape. The NS program model (sessions based on local cultural values that are associated with cultural practices) can be used in AI/AN communities and remain culturally grounded with proper community refinement and specificity.

### Refinement of the NS program

The SRPMIC CAB started by reviewing the original NS curriculum. They kept all of the existing values but added three values that they wanted to pass on to the adolescents of the SRPMIC: responsibility, teamwork, and service. The CAB then identified local cultural practices that were associated with each cultural value; each cultural practice can be associated with multiple values. However, each program session focuses on one cultural value and one associated practice for the sake of organization and reproducibility in an after‐school format. CAB members then identified local cultural practitioners who would be comfortable leading each of the NS sessions.

Finally, CAB members decided on the appropriate order of each session depending on the time of year and specific cultural practice. Although the session order is interchangeable, the CAB promoted *Language* as the first session. This allows the participants to learn how to properly introduce themselves in their traditional language, a task that is considered respectful in many AI/AN communities, and to practice their introductions each week with a knowledgeable leader and with their peers for the duration of the program. The development of the NS curriculum was an iterative process that took place over a six month period during monthly meetings with detailed feedback from CAB members and additional input from session leaders, community members, and tribal council members. Table 2 shows the NS program components.

**Table 2:** Native Spirit session outline

Cultural value	Cultural practice	Description
Language	Introduce yourself	Participants will be able to introduce themselves using their Indigenous language and recognize their Indigenous ancestry
Creation stories	Storytelling	Participants will be able to describe community creation stories, relate lessons from creation stories to having strength in cultural identity, and be able to discuss the importance of traditional archetypes like the sun, moon, and stars
History	Visit historical site	Participants will be able to recognize the importance of a site that is culturally significant to the community and describe the current history that has shaped the community
Responsibility^a^	Planting seeds	Participants will be able to describe the importance of personal responsibility and how it relates to being a leader in the community.
Respect	Harvesting and gathering	Participants will be able to articulate the importance of having respect for the environment, community, and self. Understand the importance of sustainability in the desert and beyond and be able to classify the various desert plants and how they are used (as medicine, for dyes, etc.)
Community	Traditional songs and dance	Participants will be able to describe the importance of community and the role it plays in our lives and participate in traditional community traditions like traditional songs and dance
Teamwork^a^	Traditional outdoor games	Participants will be able to describe the importance of working together in a positive way as peers and community members and relate traditional games and physical activity with the value of teamwork and interrelatedness
Patience	Traditional and modern art	Participants will be able to describe the importance of being patient in all aspects of life and participate in traditional artistry to demonstrate how being patient can allow us to create beautiful and meaningful things
Traditions	Cooking a meal	Participants will be able to relate cultural traditions of cooking with contemporary family traditions and participate with parents/guardians in cooking/eating a simple traditional meal
Growth	Coming of age	Participants will be able to articulate the roles and responsibilities of Indigenous adolescents and describe the traditional coming of age ceremonies in the community
Service^a^	Service for community	Participants will be able to recognize the importance of service to the community and recognize the different ways to provide service to the community
Cultural identity	Life away from home	Participants will be able to discuss strategies for maintaining a cultural identity on and off the reservation
Recognition	Ceremony of recognition	Participants will be able to reflect on the cultural values and activities experienced during the Native Spirit program

^a^ Values/practices that were added through Salt River Pima‐Maricopa Indian Community refinement.

### Implementation strategy

#### Logistics

NS was developed for use by local youth‐serving ASPs with middle school and high school‐aged adolescents. Each NS session ran for 1.5 hours each week except in weeks with a conflicting holiday or school event (e.g., spring break, fall break). Each NS session, of the 13 sessions available, is meant to be led by one to two people who are cultural knowledge holders and leaders in the SRPMIC. Middle and high school adolescents in the community are combined and regularly interact in ASPs.

#### Session leaders

Recruiting cultural practitioners to serve as session leaders is integral to presenting culturally grounded interventions (Okamoto, Helm, et al., 2014; Okamoto, Kulis, et al., 2014). The intergenerational transmission of knowledge provides a direct link for adolescents to learn and grow in their cultural identity. CAB members identified and helped me contact community members to lead each program session, and guided the evaluation plan. I held individual, 30–60 minute, training meetings for each session leader to discuss the overall program goals, and specific session objectives and details. I gave each session leader a description of the cultural value and associated practice they were to address, along with a set of two to three objectives for the session. Session leaders were free to create the session according to their interpretation of the value and practice while staying true to the objectives. This supports the idea that some amount of session leader flexibility and adaptability is necessary to meet local and individual needs (Breitenstein et al., 2010). Session leaders received dinner at each session to compensate for their time and expertise, and they were also invited to the recognition ceremony dinner at the conclusion of the program.

#### Evaluation strategy

The evaluation plan for the NS program was based on the principles of Indigenous evaluation and focuses on community priorities for adolescent health. Indigenous evaluation centralizes tribal context and Indigenous knowledge while building capacity in the community. Indigenous researchers have long called for research and evaluation that is grounded in Indigenous values (Grover & RMC Research Corporation, 2010; LaFrance & Nichols, 2008). While the implementation of Indigenous evaluation may be different depending on the context, the core values remain constant along with the use of culturally valid measures. Culturally valid measures include oral measures, elder review, and community contributions to evaluation (Grover & RMC Research Corporation, 2010). The NS program attended to the relationship between the program and community, while honoring tribal sovereignty, by disseminating evaluation results according to the CARE (collective benefit, authority for control, responsibility, ethics) principles for Indigenous Data Governance set forth by the Global Indigenous Data Alliance (Research Data Alliance International Indigenous Data Sovereignty Interest Group, 2019). We designed the evaluation to respect the importance of place for AI/AN communities, such that this specific iteration of the NS program cannot be directly transferred to another place without refinement. For example, implementing and evaluating the NS program that was refined for the SRPMIC would be inauthentic and unethical for a community on the East Coast because the cultural values, practices, and teachings are specific to SRPMIC. Additionally, the NS study recognized personal sovereignty by using multiple ways to measure accomplishment. This fits within a soft systems methodology that emphasizes the presence of multiple, valid perspectives and social constructions of reality and also encourages the development of individual strengths and communication styles (Foster‐Fishman & Behrens, 2007; LaFrance & Nichols, 2008).

The evaluation plan to determine the impact of the NS program consisted of several different data sources including pre‐ and posttest surveys and qualitative semi‐structured interviews with a subset of the participants. Evaluation measures were reviewed and approved by the CAB. All participants were administered an in‐person survey before the first session and after the last session. The survey measures constructs of importance for the SRPMIC including demographic information, cultural identity, self‐esteem, and resilience. The purpose of the interviews was to further examine participant experiences and their thoughts, feelings, and attitudes on cultural identity, resilience, and self‐esteem after participation. Data analysis contains direct community contributions by including members of the SRPMIC in the analysis design, process, and dissemination. Mikah Carlos (coauthor and member of the CAB) assisted with qualitative data analysis, and as a member of the SRPMIC, with expertise in statistical methods, assisted with quantitative analysis. Findings from the Fall 2019 iteration of the NS program are forthcoming.

## DISCUSSION

This article describes the development, implementation strategy, and evaluation strategy of the culturally grounded NS program with AI/AN adolescents who are members of the SRPMIC. Throughout this discussion, we offer several takeaways that can be used by public health professionals who want to engage in similar partnerships and research. The use of cultural engagement as a form of PYD is the main focus and strength of the NS program. AI/AN communities share a common experience of traumatic loss of cultural practices, language, life, and land (Running Bear et al., 2017, 2018). Many AI/AN communities continue to feel the negative impacts of cultural loss through each passing generation (Soto et al., 2015). The current movement to increase cultural engagement for adolescent AI/ANs is one way to promote health and prevent negative impacts of intergenerational trauma (Snowshoe et al., 2016). Research has shown that adolescents who are culturally engaged experience better health and educational outcomes (Smokowski et al., 2014; Tsethlikai & Rogoff, 2013).

The ASP format of NS offered important advantages for dissemination. Rather than establishing interventions separate from school or after‐school spaces, researchers should partner with local ASPs. ASPs are an accessible form of PYD that often have pre‐existing structures and resources for adolescents to travel from their schools to the programs and then to their homes. Additionally, ASPs do not have the same restraints on time and curriculum that exist for school‐based programs. The BGC, specifically, has 200 Clubhouses in Indian Country throughout the United States so there is tremendous opportunity to provide community‐specific cultural programming for AI/AN adolescents (Lekuton & Zimmerman, 2003). Additionally, AI/AN communities implement their own innovative ASPs that are not described or reported in gray literature or peer‐reviewed literature. For example, the SRPMIC runs multiple ASPs outside of the BGC that appeal to the local youth, including an ASP that is specifically for teenage girls and another that is combined for teenage girls and boys. The SRPMIC recognizes the importance of offering a safe after‐school space for youth but is not recognized for this in the scientific literature that marginalizes community knowledge.

The SRPMIC has a robust ASP selection, but unfortunately, this is not always the case. Creating a template for the NS program that can be refined to be community‐specific gives interested communities an advantage in the program implementation process, and cuts down on the administrative experimentation that is necessary when developing a program. The NS ASP is responsive to the unique community contexts and may be refined by other tribal communities and organizations throughout the United States. With further evaluation, the NS program model can be used to advocate for cultural engagement as a form of PYD and health promotion. Centering AI/AN cultural values and practices as a form of health promotion has been missing in AI/AN health research thus far.

### Challenges and limitations

The main challenge of developing and implementing the NS program is ensuring its sustainability and future implementation. This is possible within community settings that serve as mediators between risk and positive outcomes (O'Donnell et al., 1993; Sonn & Fisher, 1998). The NS program activity settings (people, norms, values, and systems of meaning) are nested within the SRPMIC youth services department that promote adolescent well‐being. Challenges exist at the activity setting level (O'Donnell et al., 1993). Although the SRPMIC and the BGC expressed a desire to start a cultural engagement program, all community members involved in the refinement and implementation held full‐time jobs that were their first priority. Without a dedicated program coordinator and funding stream, NS may be discontinued. The first half of the Spring 2021 NS iteration was attended by youth who were enrolled in other programs. Spring 2021 and Fall 2021 iterations were suspended due to the COVID‐19 pandemic, but plans are in place to start NS in Spring 2022. There are three different ASPs in the SRPMIC that have a consistent meeting time and place to give the members an opportunity to participate in NS. Although this approach could ensure community involvement and serve a higher number of adolescents, it could also result in a void of leadership to take direction in contacting session leaders and ensuring continuous evaluation.

### Recommendations for sustainability

The initial success of the NS program was due to the large community investment, from various tribal departments, youth‐serving programs, and individual community members. Our recommendations for future refinement, implementation, and evaluation of NS include ensuring that there is adequate community investment and engagement for sustainability. This can be accomplished by disseminating information on the goals and successes of previous NS program iterations. We advise that one tribal community or organization be responsible for the program with input and support from the greater community, including funding in program, government, and research budgets to compensate cultural knowledge holders (session leaders) for their time and expertise. Additionally, we must emphasize that the common goal of increasing cultural engagement in AI/AN communities is to ensure the proliferation of cultural values and practices. Examples of recently developed culturally grounded programs include the Elders' Resilience Curriculum, Ho'ouna Pono, and Qungasvik (Cwik et al., 2019; Okamoto, Helm, et al., 2014; Okamoto, Kulis, et al., 2014; Rasmus et al., 2019; Walters et al., 2020). Shared community investment and a sense of responsibility with a common goal will help the NS program remain sustainable even through changes in program leadership and funding opportunities.

Another recommendation is to ensure there is a continuous effort to evaluate the NS program using Indigenous evaluation methods. The academic–community partnership was vital for developing the NS program and evaluation plan. CAB members who indicated a desire for this program had been identified in the past; however, there was always a need for extra assistance and resources to create a well‐documented program. The academic–community partnership produced some of the needed resources, including administrative support and experience in the creation of ASPs, and the use of evaluation methods. SRPMIC has been committed to sustaining the program since it began. It has coordinated efforts by SRPMIC tribal ASP for adolescents, social services, and the teen girls' program to share the responsibility of offering NS. As a result, more adolescents in the SRPMIC will have access to the program and resources for several years.

At this point, NS is fairly small; it has been implemented twice in two tribal communities and has reached approximately 30 adolescents. Standards in prevention science marginalize Indigenous approaches by requiring sample sizes that are unrealistic for culturally grounded interventions (Flay et al., 2005). Researchers address this issue by conducting multiyear cohort studies and wait‐list control designs (Fok et al., 2015; Okamoto et al., 2019). Community‐centered formative and summative data must be collected to establish the effectiveness and to help identify components that impact AI/AN adolescent health and well‐being. Establishing an evaluation plan before implementing the NS program will allow communities to be prepared for data collection throughout the program. Additionally, we recommend that evaluation includes measures of health and well‐being, such as self‐esteem, resilience, cultural identity, stress, and other measures of behavioral and mental health status. The connection between cultural identity for AI/AN adolescents and health has not been well‐established (Kagawa Singer et al., 2016). Collecting health outcome data will help AI/AN communities contribute to developing an Indigenous prevention science (Okamoto, Helm, et al., 2014; Okamoto, Kulis, et al., 2014).

## CONCLUSIONS

An academic–community partnership successfully developed and implemented a culturally grounded ASP for AI adolescents living on an urban‐based reservation in the Southwest. Documentation of the development and implementation of the NS program contributes to an Indigenous prevention science that examines the impacts of AI/AN culture on adolescent health and well‐being. The accessible and widely used format of ASPs provides opportunities for disseminating the culturally grounded and strengths‐based NS program to BGCs, ASPs in Indian Country, and urban AI/AN organizations across the country.
